# VISUAL IMPAIRMENT AND ENGAGEMENT IN COGNITIVELY STIMULATING ACTIVITIES

**DOI:** 10.1093/geroni/igz038.2432

**Published:** 2019-11-08

**Authors:** Bonnielin Swenor, beatriz Munoz, Eleanor M Simonsick

**Affiliations:** 1 The Wilmer Eye Institute, Johns Hopkins University School of Medicine, Baltimore, Maryland, United States; 2 The Wilmer Eye Institute, Johns Hopkins School of Medicine, Baltimore, Maryland, United States; 3 Longitudinal Studies Section, Intramural Research Program, National Institute on Aging, Baltimore, Maryland, United States

## Abstract

We examined the relationship between visual impairment (VI) and engagement in cognitively stimulating activities using data from 924 participants in the Cognitive Vitality Sub-Study of the Health ABC Study. At Year 3 (baseline for these analyses), vision was assessed as: visual acuity (VA), contrast sensitivity (CS), and stereo acuity (SA). Participation in cognitively stimulating activities was determined based on responses to 12 questions (administered at Years 3, 5, 7, and 9) assessing frequency of participation ranging from none to daily. We calculated the total number of activities engaged in at least monthly. In cross-sectional analyses adjusted for age, race, and sex, impaired VA (≤20/40, 8%), CS (<1.55, 5%), and SA (<80 secs arc, 29%) was associated with participation in fewer cognitive activities (β=-0.54, 95% CI:-1.06, -0.03; β=-0.59, 95% CI:-0.12, 0.06; β=-0.40, 95% CI:-0.81, -0.18, respectively). Longitudinally, change per year in the number of activities differed by baseline participation levels. Those participating in ≥5 activities at baseline (population median) had a significant decline in the number of activities, irrespective of VI status. However, for those participating in <5 activities at baseline, the increase in these activities tended to be lesser in the VI than in non-VI groups, and for SA this increase was significantly lower for the impaired group (βimpaired=0.004; 95% CI:-0.05, 0.05; βnot-impaired=0.06; 95% CI: 0.03, 0.10; time x SA interaction p=0.0496). These data indicate that older adults with VI participate in fewer cognitive activities and the change in participation over time differs from than those without VI.

